# Aquaporin7 plays a crucial role in tolerance to hyperosmotic stress and in the survival of oocytes during cryopreservation

**DOI:** 10.1038/srep17741

**Published:** 2015-12-04

**Authors:** Ya-Jing Tan, Xue-Ying Zhang, Guo-Lian Ding, Rong Li, Li Wang, Li Jin, Xian-Hua Lin, Ling Gao, Jian-Zhong Sheng, He-Feng Huang

**Affiliations:** 1 Center of Reproductive Medicine, the International Peace Maternity and Child Health Hospital, School of Medicine, Shanghai Jiao Tong University, Shanghai, China; 2 Institute of Embryo-Fetal Original Adult Disease Affiliated to Shanghai Jiao Tong University School of Medicine, Shanghai Jiao Tong University, Shanghai, China; 3 The Key Laboratory of Reproductive Genetics, Ministry of Education (Zhejiang University), Hangzhou, China; 4 Department of Pathology & Pathophysiology, School of Medicine, Zhejiang University, Hangzhou, China

## Abstract

Hyperosmotic stress may induce apoptosis of different cells. However, oocytes show tolerance to osmotic stress during cryopreservation by vitrification, which is an assisted reproductive technique. The underlying mechanism is still not understood. Here, we demonstrated that hyperosmosis produced by high concentrations of cryoprotectants, including DMSO, ethylene glycol and sucrose, significantly upregulated the protein levels of aquaporin (AQP) 7, but not AQP3 and AQP9, in mouse oocytes. Knockdown of AQP7 expression by siRNA-injection significantly reduced the survival of oocytes after vitrification. In oocytes, AQP7 was shown to bind with F-actin, a protein involved in almost all biological events. Moreover, we found that hyperosmosis could upregulate the phosphorylation levels of CPE-binding protein (CPEB) and Aurora A. Inhibition of the PI3K and PKC pathways blocked the hyperosmosis-induced upregulation of AQP7 and the phosphorylation of CPEB and Aurora A in oocytes. In conclusion, hyperosmosis could upregulate the expression of AQP7 via Aurora A/CPEB phosphorylation mediated by the PI3K and PKC pathways, and upregulation of AQP7 plays an important role in improving of tolerance to hyperosmotic stress and survival of oocytes during cryopreservation by vitrification.

Human oocyte cryopreservation is an important technology in assisted reproduction, and may help to preserve the future fertility of women who face cancer/extirpative therapy or who want to extend their childbearing years. It also avoids the many legal and ethical issues associated with embryo freezing^1^. Oocyte cryopreservation also provides the possibility of saving oocyte to build a human oocyte bank^2^. Cryopreservation procedures involve several steps, including the addition of cryoprotectant and its removal from cells, and, cooling and warming. There are two methods for oocyte cryopreservation: the traditional slow cooling of oocytes and the vitrification of oocytes. Traditional cryopreservation of oocytes by slow cooling methods has been shown to be ineffective because the oocytes are more sensitive to chilling-induced injury; therefore vitrification has been suggested as the best alternative^3^.

During cryopreservation by vitrification, an oocyte is placed in a hypertonic solution containing 1–2 mol cryoprotectant. The cell initially shrinks rapidly in response to the high extracellular osmolarity, and extracellular cryoprotectants exchange with intracellular water until the cryoprotectant permeates the cell with water at a fixed osmolarity. Such insults compromise oocyte viability and developmental capacity^4^. Therefore, tolerance to osmotic stress may determine the survival of oocytes during cryopreservation by vitrification. Ethylene glycol (EG) and DMSO are frequently used as penetrating cryoprotectants, and sucrose is used as a non-penetrating cryoprotectant. The permeability of the plasma membrane to water and cryoprotectants is important for the tolerance of cells to osmotic stress^5^,^6^.

Aquaporins (AQPs), members of a superfamily of transmembrane channel proteins, are ubiquitous in all domains of life^4^,^7^,^8^. Previous studies have shown that AQP3 and AQP7 are expressed in mature human^9^ and mouse oocytes^10^,^11^,^12^. AQP3 and AQP7 are involved in the aquaglyceroporin subtype of aquaporins, which are permeable not only to water but also to small neutral solutes^13^. In Xenopus oocytes, AQP7 shows permeability to water, glycerol, and urea^14^,^15^,^16^. Our previous study demonstrated that cryoprotectants, including DMSO and EG, might upregulate AQP7 protein expression in mouse oocytes during cryopreservation^12^. However, the underlying mechanism is unclear.

The oocyte is a unique cell whose life cycle is characterized by alternating periods of active meiotic progression and long periods of meiotic arrest. Gene expression during oocyte maturation, fertilization and early embryo development, until zygotic gene activation, is mainly regulated by timely translational activation of specific maternally derived mRNAs, which are accumulated in the oocyte before the first start of meiosis^17^,^18^. A primary pathway that mediates mRNA storage involves cytoplasmic polyadenylation element-binding protein (CPEB), which binds cytoplasmic polyadenylation element (CPE) at the 3′-untranslated end of mRNAs^19^. When the upstream protein Aurora A is activated by phosphorylation, the activated Aurora A phosphorylates CPEB. When phosphorylated CPEB (pCPEB) combines with a split polyadenylation specificity factor (CPSF) and polyA polymerase (PAP), which increases the length of polyA tails on mRNAs, translation is initiated^18^. However, whether the osmotic stress alters gene expression via the Aurora A and CPEB phosphorylation pathway is unknown.

In the present study, we found that a hyperosmotic cryoprotectant solution containing EG, DMSO and sucrose, respectively, increase expression of AQP7 in oocytes, but not the expression of AQP3 and AQP9, which are the same subtype as AQP7. The decreased expression of AQP7 significantly reduced the survival of oocytes after vitrification. AQP7 was shown to bind with F-actin. In response to hyperosmotic stress, the phosphorylation of CPEB and Aurora A were significantly increased. Moreover, we found that PI3K and PKC inhibitors significantly blocked the effects of the hyperosmotic EG solution on the upregulation of AQP7 and on the phosphorylation of CPEB and Aurora A expression in oocytes. These results provide novel insights into the mechanisms of AQP7 in tolerance to hyperosmotic stress during oocyte cryopreservation.

## Results

### Hyperosmosis induces upregulation of AQP7, not AQP3 and AQP9, protein levels in mouse oocytes

**Figure 1:**
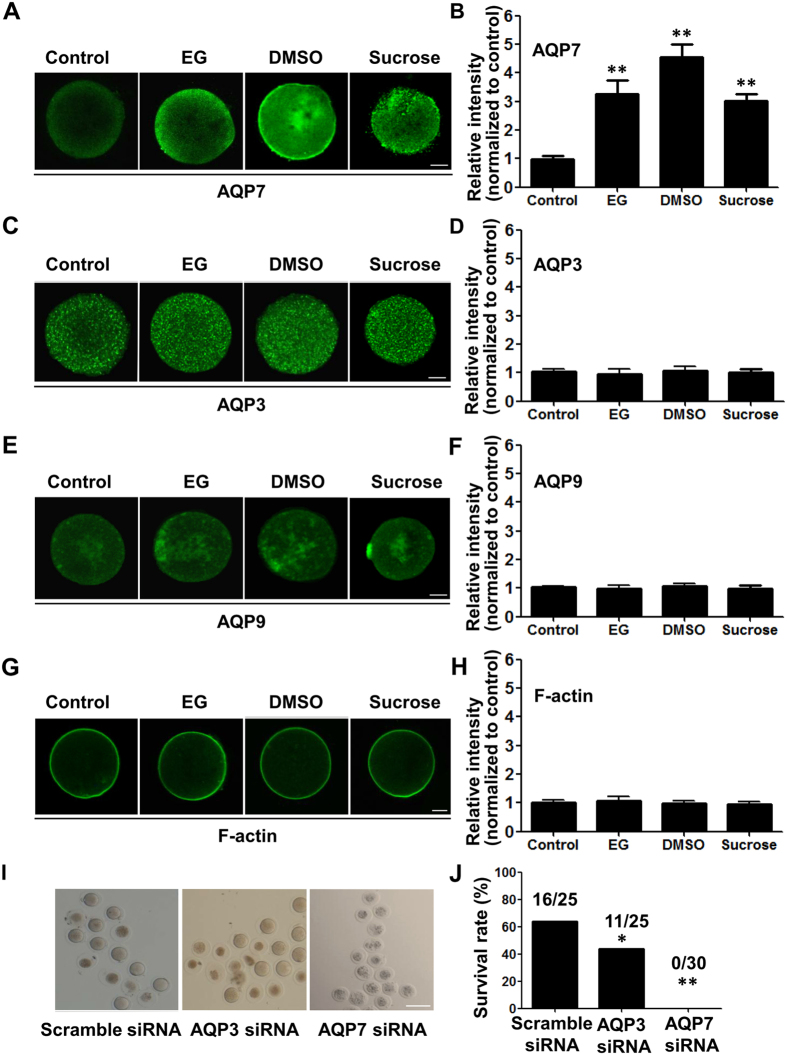
Hyperosmotic cryoprotectant solutions increased AQP7, but not AQP3 and AQP9 expression in mouse oocytes. (**A**) Immunofluorescence analysis of AQP7 expression in mouse oocytes in the presence of 8% EG, 9.5% DMSO and 0.5 M sucrose, respectively. (**B**) Summary data of the immunofluorescence analysis in (**A**). (**C**) Immunofluorescence analysis of AQP3 expression in mouse oocytes treated as in (**A**). (**D**) Summary data of the immunofluorescence analysis. (**E**) Immunofluorescence analysis of AQP9 expression in mouse oocytes treated as in (**A**). (**F**) Summary data of the immunofluorescence analysis. (**G**) Immunofluorescence analysis of F-actin expression in mouse oocytes treated as in (**A**). (**H**) Summary data of the immunofluorescence analysis. Scale bar (**A**–**H**), 20 μm. Data in (**A**–**H**) are presented as the mean ± SE, n ≥ 6, ** *P* < 0.01 compared with the corresponding control (Student’s *t* test). (**I**) Image of thawed oocytes transfected with scramble siRNA, AQP3 siRNA or AQP7 siRNA after cryopreservation for 48 h with EG as the cryoprotectant. Scale bar, 100 μm. (**J**) Survival rate of the oocytes after thawing for 2 h. *P < 0.05 and **P < 0.01 compared to the corresponding control (Chi-square test).

Our previous studies have shown that human and mouse oocytes expressed AQP3 and AQP7, two members of the aquaglyceroporin family^9^,^12^. In this study, we examined AQP9, another member of the aquaglyceroporin family, in mouse oocytes, and found that mouse oocytes also express AQP9 (Supplementary Fig. S1). To examine whether hyperosmotic stress induced alterations of aquaglyceroporin expression in oocytes, we treated mouse oocytes with a high concentration of two penetrating cryoprotectants, EG and DMSO, and a non-penetrating cryoprotectant, sucrose, respectively. We found that all three hyperosmotic cryoprotectant solutions could upregulate AQP7 protein expression in oocytes (Fig. 1A,B). However, these hyperosmotic cryoprotectant solutions did not upregulate AQP3 and AQP9 protein expression in mouse oocytes (Fig. 1C–F). On the other hand, expression of F-actin in oocytes was not changed after treatment with the EG, DMSO or sucrose solutions (Fig. 1G,H). The results suggest that hyperosmotic stress may selectively upregulate AQP7 expression in mouse oocytes, and AQP7 may play a main function in water and cryoprotectant transport during oocyte cryopreservation.

To examine the functions of AQP7 in oocytes during cryopreservation, we knocked down AQP7 in mouse oocytes by injecting them with siRNA targeting AQP7 at the GV stage. Expression levels of AQP7 mRNA and protein in oocytes injected with AQP7 siRNA were significantly lower than in oocytes injected with scrambled siRNA (Supplementary Fig. S2). Then we performed the cryopreservation by vitrification using EG as the cryoprotectant. After thawing, the oocytes injected with AQP7 siRNA were dark in colour and had shrunk (Fig. 1I). The survival rate of the oocytes injected with AQP7 siRNA was 0%, which was significantly lower than that of the oocytes injected with scrambled siRNA (64%) (Fig. 1J, *P* < 0.05, Chi-square test). On the other hand, we treated mouse oocytes with AQP3 siRNA and found that the survival rate of these oocytes was 44%, which was lower than that of the oocytes injected with scrambled siRNA, but higher than the AQP7-knockdown oocytes after thawing (Fig. 1I). These results indicate that AQP7 is a main water channel involved in tolerance to hyperosmotic stress in oocytes during cryopreservation.

### Hyperosmosis induced redistribution of AQP7 in the cell membrane

**Figure 2:**
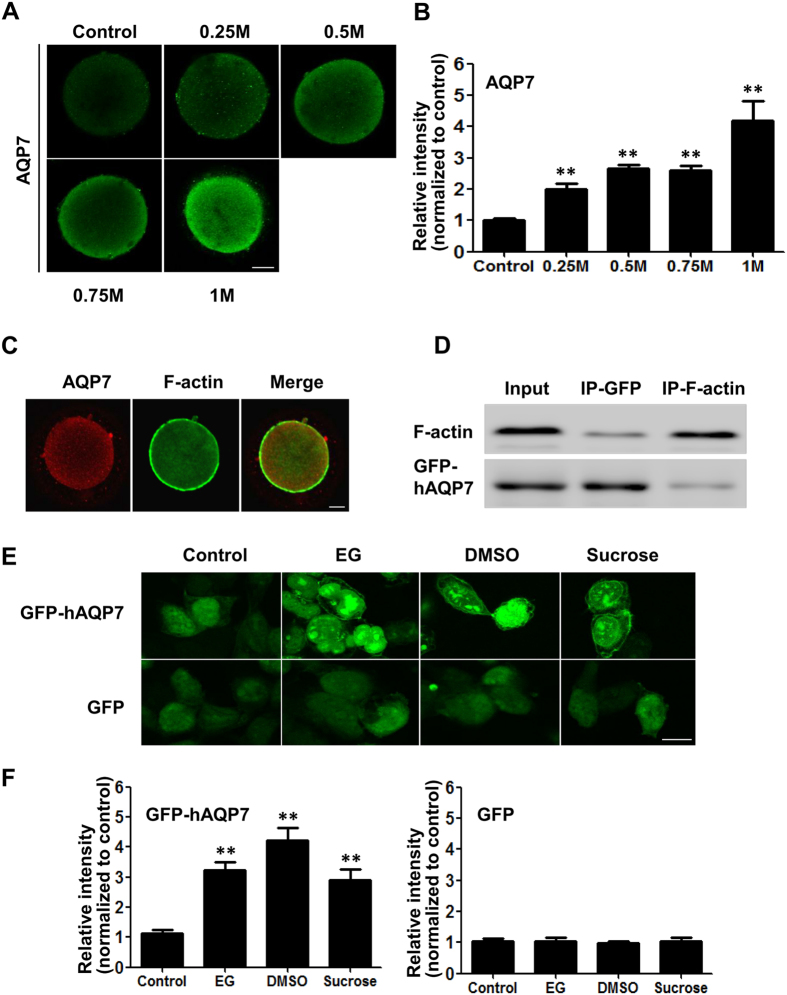
Hyperosmosis induced redistribution of AQP7 in the cell membrane. (**A**) Immunofluorescence analysis of AQP7 expression in mouse oocytes in the presence of 0.25 M, 0.5 M, 0.75 M and 1 M sucrose, respectively. (**B**) Summary data of the immunofluorescence analysis (n ≥ 5). (**C**) Immunofluorescence analysis of AQP7 and F-actin colocalization in mouse oocytes (red: AQP7; green: F-actin). Scale bar (**A**–**C**), 20 μm, (**D**) Co-immunoprecipitation of AQP7 and F-actin. IP, immunoprecipitation. (**E**) Green fluorescent protein (GFP) intensity in 293FT cells transfected with the GFP-hAQP7 fusion protein expression plasmid or with the GFP vector alone in the presence of 8% EG, 9.5% DMSO, and 0.5 M sucrose, respectively. Scale bar, 10 μm. (**F**) Summary data of the immunofluorescence analysis (calculated for 20 cells for each condition obtained from three independent experiments). Data are presented as the mean ± SE, **P < 0.01 compared to the corresponding control.

It has been shown that osmotic pressure can stimulate aquaporin gene expression in rat astrocytes^20^. We treated mouse oocytes with different concentrations of a non-penetrating cryoprotectant, sucrose, which resulted in different osmotic pressures, and found that gradual increases in osmotic pressure induced gradual upregulation of AQP7 levels in the cell membrane (Fig. 2A,B). As a cytoskeleton protein, F-actin has been reported to be involved in the trafficking of many intracellular proteins^21^. To examine whether F-actin might facilitate the translocation of AQP7 from the cytoplasm to the cell membrane, we observed the interaction between AQP7 and F-actin. Immunofluorescence analysis showed co-localization of AQP7 with F-actin in oocytes (Fig. 2C, supplementary Fig. S3). This co-localization was further confirmed by a co-immunoprecipitation experiment in 293FT cells expressing GFP-hAQP7. Immunoblot analysis showed that F-actin was present in the GFP-hAQP7 immunoprecipitate (Fig. 2D). In the converse experiment, GFP-hAQP7 was present in the F-actin immunoprecipitate (Fig. 2D). This result suggests that AQP7 proteins may be transported by F-actin from the cytoplasm to the cell membrane, where AQP7 facilitates water and cryoprotectant transport and improves osmotic balance.

To confirm that hyperosmotic cryoprotectant solutions had a direct effect on AQP7 expression in the cells, we constructed a GFP-hAQP7 fusion protein expression plasmid and transfected this plasmid into 293FT cells. Treatment of GFP-hAQP7 plasmid-transfected 293FT cells with EG, DMSO or sucrose induced a significant increase in GFP fluorescence intensities (Fig. 2E,F). Additionally, we noticed that the fluorescence intensities in the cell membrane also increased (Fig. 2E), suggesting that hyperosmotic stress could induce the translocation of more AQP7 proteins to the cell membrane where they perform their function of facilitating water and cryoprotectant transport. However, treatment of 293FT cells transfected with a plasmid that expressed GFP alone with DMSO, EG or sucrose did not alter GFP fluorescence intensities (Fig. 2E,F).

### Hyperosmosis induced CPEB phosphorylation in oocytes

**Figure 3:**
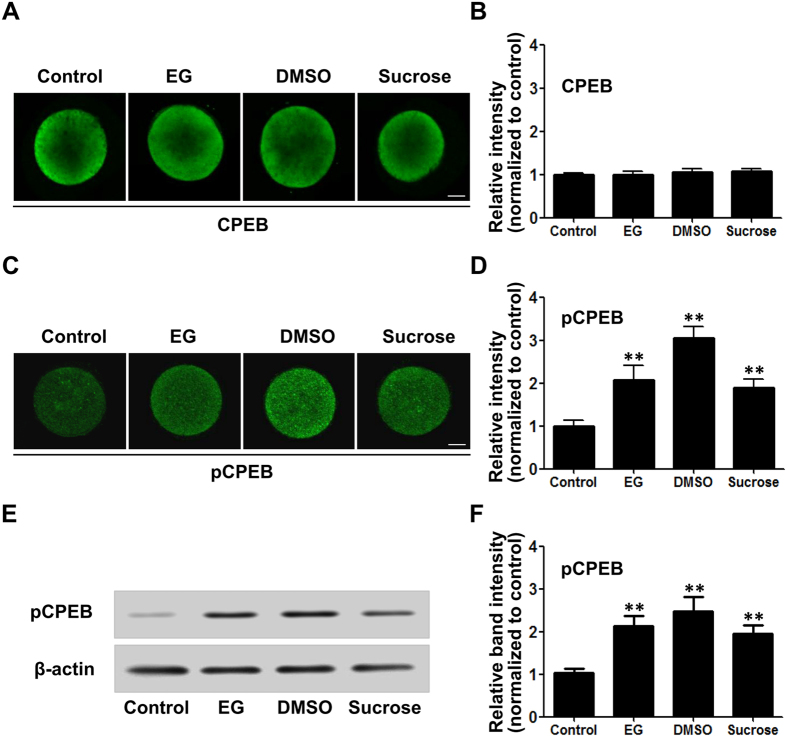
Hyperosmotic cryoprotectant solutions significantly increased CPEB phosphorylation in mouse oocytes. (**A**) Immunofluorescence analysis of total CPEB protein level in the presence of 8% EG, 9.5% DMSO, and 0.5 M sucrose, respectively. (**B**) Summary data of the immunofluorescence analysis. (**C**) Immunofluorescence analysis of phosphorylated CPEB (pCPEB) protein level in mouse oocytes treated as in (**A**). (**D**) Summary data of the immunofluorescence analysis (n ≥ 6). (**E**) Western blotting analysis of phosphorylated CPEB (pCPEB). (**F**) Summary data of Western blotting analysis (n = 3). Data are presented as the mean ± SE. **P < 0.01 compared to the corresponding control. Scale bar, 20 μm.

CPEB is a translational regulatory sequence-specific RNA-binding protein that controls oocyte development. It binds with mRNA to prevent translation. When it is phosphorylated, the mRNAs are released and translated. After treating mouse oocytes with a hyperosmotic solution containing EG, DMSO or sucrose, we did not detect a significant change in total CPEB protein levels in any of the groups (Fig. 3A,B). However, the immunofluorescence intensities of phosphorylated CPEB were significantly increased in the three groups (Fig. 3C,D). Western blotting showed the same results (Fig. 3E,F).

### Hyperosmosis induced Aurora A phosphorylation in oocytes

**Figure 4:**
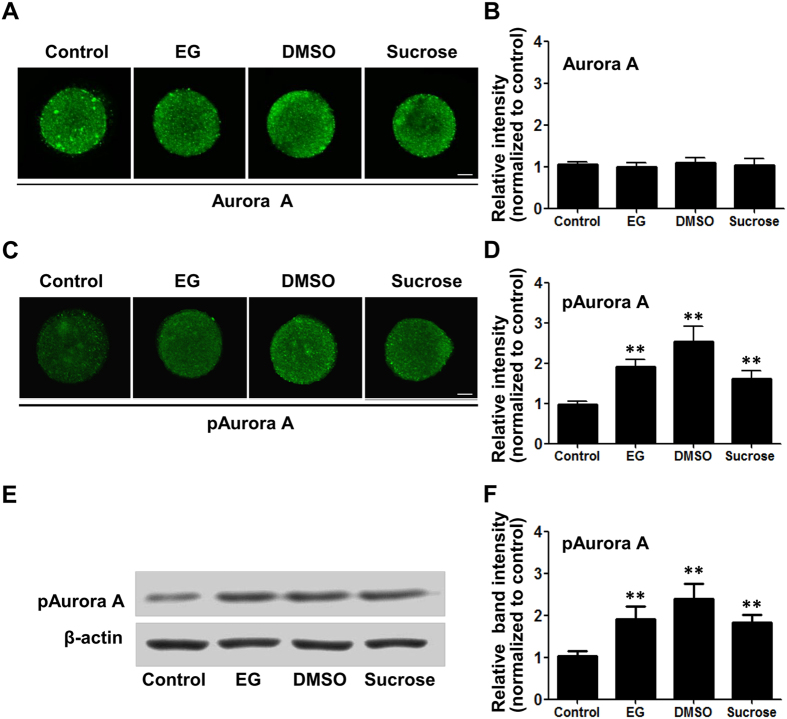
Hyperosmotic cryoprotectant solutions significantly increased phosphorylation of Aurora A in mouse oocytes. (**A**) Immunofluorescence analysis of total Aurora A protein level in the presence of 8% EG, 9.5% DMSO, and 0.5 M sucrose, respectively. (**B**) Summary data of the immunofluorescence analysis. (**C**) Immunofluorescence analysis of phosphorylated Aurora A (pAurora A) protein level in mouse oocytes treated as in (**A**). (**D**) Summary data of the immunofluorescence analysis. Data are presented as the mean ± SE, (n ≥ 6). (**E**) Western blotting analysis of of pAurora A. (**F**) Summary data of Western blotting analysis (n = 3). Data are presented as the mean ± SE. **P < 0.01 compared to the corresponding control. Scale bar, 20 μm.

Oocyte maturation requires Aurora A-catalysed CPEB serine 174 phosphorylation and CPE-dependent cytoplasmic polyadenylation^22^. Aurora A undergoes several phosphorylation events, which are cell-cycle-controlled. T288 phosphorylation of Aurora A allows this kinase to be activated^23^. When mouse oocytes were treated with hyperosmotic EG, DMSO or sucrose solutions, the levels of phosphorylated Aurora A (pAurora A) protein in the oocytes was significantly increased in the three groups (Fig. 4C,D), although there was no significant change in the total Aurora A protein level (Fig. 4A,B). Western blotting showed that pAurora A increased significantly in the three groups (Fig. 4E,F).

### PI3K and PKC pathways mediate the upregulation of the Aurora A/CPEB phosphorylation induced by hyperosmosis

**Figure 5:**
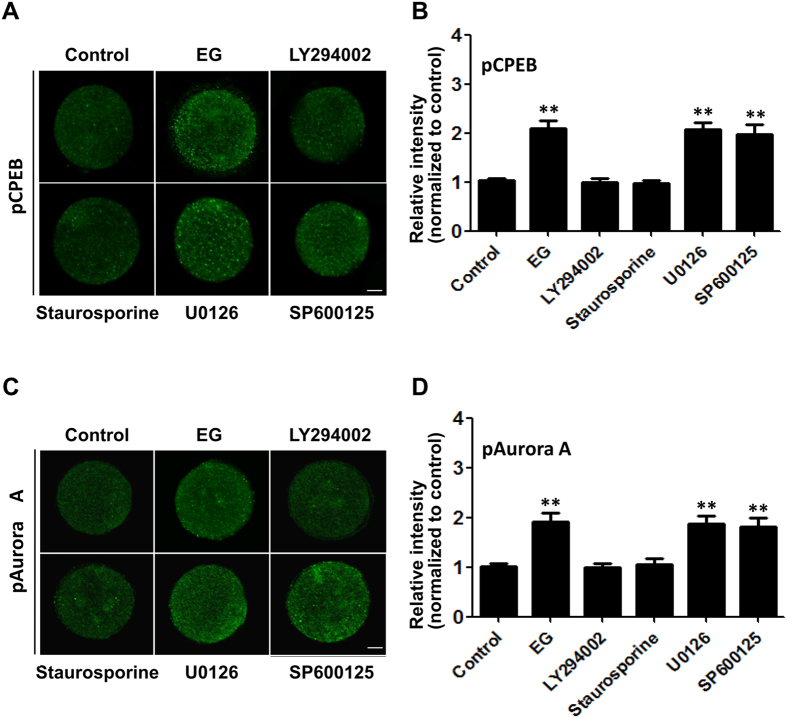
PI3K and PKC inhibitors significantly blocked the upregulation of CPEB and Aurora A phosphorylation levels. (**A**) Oocytes were pretreated with LY294002, staurosporine, U0126 and SP600125, respectively, and the immunofluorescence intensities of phosphorylated levels of CPEB (pCPEB) in oocytes were analysed in the presence of 8% EG. (**B**) Summary data of the immunofluorescence analysis. (**C**) Oocytes were pretreated as in (**A**), and the immunofluorescence intensities of phosphorylated Aurora A (pAurora A) in oocytes were analysed in the presence of 8% EG. (**D**) Summary data of the immunofluorescence analysis. Data are presented as the mean ± SE (n ≥ 7). **P < 0.01 compared to the corresponding control. Scale bar, 20 μm.

To clarify the upstream signal pathway that activates Aurora A/CPEB, selective inhibitors were used to identify the signaling pathway involved. We examined hyperosmotic stress-induced CPEB and Aurora A phosphorylation in mouse oocytes that were pretreated with a PI3K inhibitor (LY294002), a PKC inhibitor (staurosporine), a MEK inhibitor (U0126) and a JNK inhibitor (SP600125), respectively. We found that the upregulation of CPEB and Aurora A phosphorylation by the hyperosmotic EG solution was blocked by the PI3K and PKC inhibitors, but not by the MEK and JNK inhibitors (Fig. 5). These results suggest that the PI3K and PKC pathways may mediate the upregulation of Aurora A/CPEB phosphorylation induced by hyperosmosis.

### Inhibition of PI3K and PKC blocked the upregulation of AQP7 expression induced by hyperosmosis

**Figure 6:**
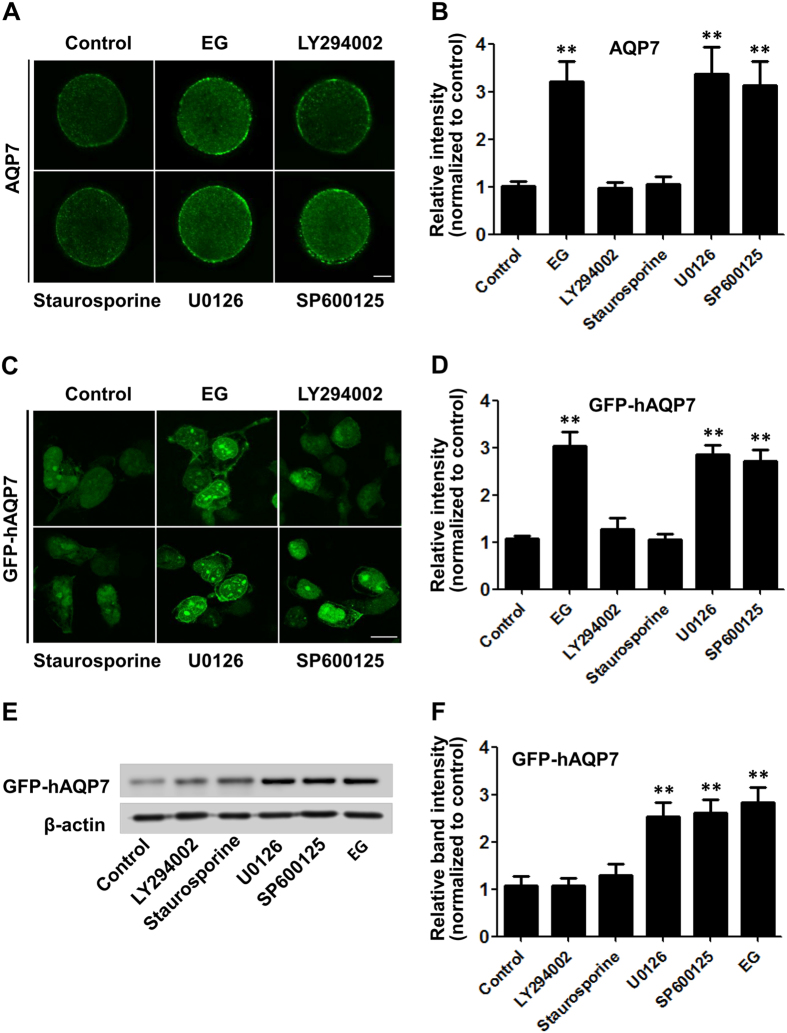
PI3K and PKC inhibitors significantly blocked the upregulation of AQP7 expression induced by the EG solution. (**A**) Oocytes were pretreated with LY294002, staurosporine, U0126 and SP600125, respectively, and the immunofluorescence intensities of AQP7 were analysed in mouse oocytes in the presence of 8% EG. Scale bar, 20 μm. (**B**) Summary data of the immunofluorescence analysis (n ≥ 7). (**C**) 293FT cells were transfected with the GFP-hAQP7 fusion protein expression plasmid and treated as in (**A**). The immunofluorescence intensities of GFP-hAQP7 were analysed in the presence of 8% EG. Scale bar, 10 μm. (**D**) Summary data of the immunofluorescence analysis (calculated for 20 cells for each condition obtained from three independent experiments). (**E**) Western blotting analysis of GFP-hAQP7 in 293FT cells treated as in (**C**). (**F**) Summary data of the Western blotting analysis (n = 3). Data are presented as the mean ± SE. **P < 0.01 compared to the corresponding control.

To clarify the signalling pathway mediating the upregulation of AQP7 by hyperosmotic stress, we examined hyperosmotic stress-induced AQP7 expression in mouse oocytes that were pretreated with LY294002, staurosporine, U0126 and SP600125, respectively. Immunofluorescence analysis showed that both LY294002 and staurosporine blocked the hyperosmotic EG solution-induced upregulation of AQP7 expression, whereas the other inhibitors had no effect (Fig. 6A,B). In addition, these inhibitors had no effects on AQP7 expression without treatment with EG solution (Supplementary Fig. S4A). We used another kind of PKC inhibitor (GF109203X) to treat oocytes. We found that the upregulated AQP7 expression which induced by hyperosmosis was also inhibited by PKC inhibitor GF109203X (Supplementary Fig. S4B). These results indicate that the hyperosmosis-induced expression of AQP7 may be mediated by the PI3K and PKC signalling pathways.

To confirm that PI3K and PKC signalling pathways mediated the hyperosmotic stress-induced upregulation of AQP7 expression, we repeated the same experiments in 293FT cells that were transfected with the GFP-hAQP7 fusion protein expression plasmid. We also found that both LY294002 and staurosporine, but not U0126 and SP600125, attenuated the fluorescence intensities of GFP-hAQP7 induced by the hyperosmotic EG solution (Fig. 6C,D). Meanwhile, Western blotting showed the same results (Fig. 6E,F).

## Discussion

Permeability of the plasma membrane to water and cryoprotectants is crucial for cell survival during cryopreservation. Aquaporins, in the cell membrane, and especially members of the aquaglyceroporin subfamily, which includes AQP3, AQP7 and AQP9, play an important role in facilitating water and cryoprotectants movement because aquaglyceroporins are permeable not only to water but also to small neutral solutes^4^,^8^,^13^. Our previous study demonstrated that AQP7 is expressed in human and mouse oocytes^9^,^12^. In the present study, we found that hyperosmosis could upregulate AQP7 expression in mouse oocytes. However, no effect of hyperosmosis on AQP3 and AQP9 expression was detected. These results suggest that hyperosmosis may selectively upregulate aquaporin expression in oocytes.

We noticed that high osmotic pressure could alter the distribution of AQP7 that was translocated from the cytoplasm to the cell membrane. When AQP7 gene expression was knocked down, the survival rate of the oocytes was significantly reduced, indicating that AQP7 is a main aquaporin subtype involved in promoting oocyte tolerance to hyperosmotic stress during cryopreservation. Osmolarity plays an important role in cellular homeostasis. A given osmotic gradient across the cell membrane induces osmotic flow. The osmotic flow can be achieved through the lipid bilayer as well as through proteins. Among these proteins, aquaporins are the main water transporters and play a crucial role in modulation of the osmotic permeability of the cell membranes^24^. Upregulation of the water transport capacity of the cell membrane with aquaporins can improve the osmotic flux generated by an osmotic gradient. The expulsion of intracellular water and a reduction in intracellular ice formation are facilitated by exposure to penetrating cryoprotectants, including propanediol, DMSO and EG, which may penetrate the cell membrane to displace water via an osmotic gradient and/or non-penetrating cryoprotectants, including sucrose, which provide a continuous osmotic gradient. Penetrating cryoprotectants also aid in balancing other intracellular solutes, which are lethal at high concentrations^3^. One of the critical steps in cryopreserving oocytes is the loading of penetrating cryoprotectants, which may result in severe osmotic perturbations and cryoprotectant toxicity depending on the specifics of the experimental protocol^25^. Although several studies of the oocyte on cryopreservation were conducted to assess the efficiency, reliability, and biosafety of cryoprotectant loading methods, these studies mainly focused on physical or chemical approaches to improve cryoprotectant loading and removal. In the present study and in previous studies, we found that hyperosmotic stress and cryoprotectants induced the upregulation of AQP7 expression in the cell membrane where aquaporins perform their functions in the facilitation of water and cryoprotectant transport. This may be one of mechanisms involved in the tolerance of oocytes to hyperosmotic stress during cryopreservation.

Translocation of aquaporins to the cell membrane plays crucial roles in oocyte maturation^26^. Rapidly accumulating evidence indicates that actin and actin-based cytoskeletal complexes are involved in protein trafficking^27^. In the kidney, AQP2 is transported to the cell membrane through actin-based microtubules formed after the new proteins are synthesized and processed^28^. The present study showed that AQP7 might bind to F-actin, suggesting that the translocation of AQP7 from the cytoplasm to the cell membrane may also depend on actin filaments to achieve better and faster completion of water and cryoprotectant exchange and maintenance of the osmotic pressure balance in oocytes.

Gene expression is regulated in several steps including transcription, post-transcriptional modification, translation, and post-translational modification. Among these processes, post-transcriptional and post-translational regulation provides cells with the ability to respond rapidly and sensitively to internal or environmental changes. When an oocyte enters the first meiotic division, transcription is stopped until the first cleavage after fertilization^17^,^18^. However, protein synthesis is still in progress, and oocytes rely on post-translational regulation, such as phosphorylation, dephosphorylation, ubiquitination, sumoylation and post-transcriptional regulation of pre-existing transcripts to precisely regulate the maturation process^29^. A large number of transcripts in cells are stored by combining with the CPEB and other proteins to form RNA protein complexes. CPEB is the critical protein that controls mRNA translation in oocytes^30^. When CPEB is phosphorylated, it is activated. Phosphorylated CPEB promotes depolymerization of RNA protein complexes, and then mRNA can be translated^31^. In our study, we found that the hyperosmotic cryoprotectant solutions upregulated the phosphorylation levels of CPEB in oocytes, suggesting that hyperosmosis might promote mRNA, including AQP7 mRNA, to be liberated and promote the translation of AQP7 mRNA. However, the hyperosmotic cryoprotectant solutions did not upregulate AQP3 and AQP9 expression. The underlying mechanism is unclear, and further studies are needed to clarify it.

CPEB and Aurora A play important roles in the regulation of gene expression in oocytes. After restarting meiosis, gene expression in oocyte is regulated by CPEB19. Aurora A is a member of a family of mitotic serine/threonine kinases, and is activated by phosphorylations at one or more sites, such as T288^23^. Phosphorylated Aurora A activates CPEB via phosphorylation of the serine 174 residue^22^. It has been shown that osmotic pressure can stimulate pressure sensors on the cell membrane to activate intracellular kinases^31^ and cause a series of physiological changes, including changes in gene expression^32^. The activated kinases may induce the phosphorylation of Aurora A. Our study demonstrates that hyperosmosis significantly increased the phosphorylation levels of both CPEB and Aurora A. On the other hand, we found that the upregulated AQP7 expression and increased phosphorylation levels of CPEB and Aurora A were blocked by PI3K and PKC inhibitors. It has been demonstrated that the hyperosmotic pressure produced by high NaCl induces phosphorylation of p85 on Y508, which is involved in the activation of PI3K^33^, and that hyperosmotic stress also increases phosphorylation of PKCδ^34^. Our results suggest that the hyperosmosis-increased upregulation of AQP7 expression may be due to the activation of the Aurora A/CPEB pathway. This pathway might be mediated by PI3K and PKC, although further studies are needed for clarification.

**Figure 7:**
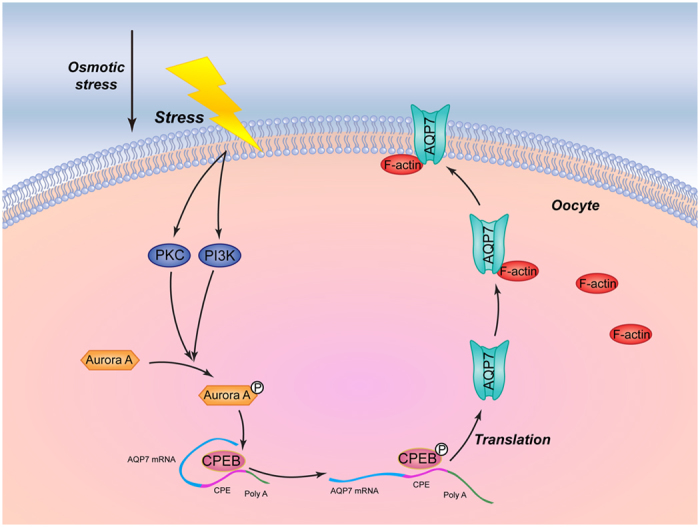
Schematic diagram depicting the potential interactions between hyperosmotic stress and the intracellular processes, including upregulation of AQP7. During the cryopreservation process, the hyperosmotic stress produced by the cryoprotectant solution activates phosphorylation of Aurora A (pAurora A) through PI3K and PKC signalling pathways. pAurora A phosphorylates CPEB, a regulator of intracellular translated protein, might result in upregulation of AQP7 protein expression. The increased AQP7 binds to F-actin and might be translocated from the cytoplasm to the cell membrane where they facilitate water and cryoprotectant transport.

Because tolerance to hyperosmotic stress is very important in cells during cryopreservation, it is important to find the underlying mechanism of tolerance to hyperosmotic stress in oocytes and other cells to improve cryopreservation protocols. Our study provides the previously undocumented insight that AQP7 in oocytes may mediate tolerance to hyperosmotic stress during cryopreservation. All together, we observed that hyperosmotic stress activated the Aurora A/CPEB pathway mediated by PI3K and PKC to upregulate AQP7 expression. F-actin may play an important role in AQP7 intracellular trafficking from the cytoplasm to the cell membrane. A schematic diagram depicting the potential interactions between these cellular processes is shown in Fig. 7. Most significantly, these observations suggest that AQP7 may be a potent candidate for predicting the quality of oocytes after cryopreservation in clinical practice.

## Materials and Methods

### Collection of mouse oocytes

All animal experiments were performed according to the appropriate guidelines for animal used approved by the Institutional Animal Care and Use Committee of the School of Medicine, Zhejiang University. C57BL/6J female mice, an inbred strain, were purchased from the Zhejiang University Animal Centre at 6 weeks of age and were housed, fed and maintained under identical conditions, with a regulated light–dark cycle (14 h light/10 h dark, starting at 6:00 A.M. each day). Superovulation was induced in 8-wee-old adult mice by intraperitoneal injection of 10 IU pregnant mare serum gonadotropin (PMSG; Sigma-Aldrich, Saint Louis, USA), followed by 5 IU hCG 48 h after the PMSG administration. Unfertilized oocytes were collected from the ampullary portions of the oviducts 14 h after the injection of hCG and were freed from cumulus cells by suspending them in human tubal fluid (HTF) containing 80 units/ml hyaluronidase (Sigma-Aldrich), followed by washing with fresh HTF. Only MII oocytes showing a normal appearance with a visible first polar body were used in this study. The number of oocytes obtained from each animal was 20. The number of animals used for this experiment was 350.

### Cell culture

The human embryonic kidney cell line (293FT) was purchased from ScienCell (Carlsbad, USA), and cultured in DMEM (Gibco, Grand Island, USA) containing 10% fetal bovine serum (Gibco). Cells were cultured at 37 °C with 5% CO_2_.

### Solutions and treatments

To evaluate the effects of cryoprotectants on the biological properties of oocytes, mouse oocytes were treated with HTF containing 8% EG, 9.5% DMSO or 0.5 M sucrose for 20 min. To evaluate the osmostic stress-dependent response, oocytes were treated with HTF containing 0.25 M, 0.5 M, 0.75 M or 1 M sucrose for 20 min. Oocytes treated with HTF alone were used as a control. After treatment, the oocytes were collected and immediately fixed at room temperature for 20 min in PBS containing 4% paraformaldehyde (PFA) for immunofluorescence analysis. Oocytes were lysed in RIPA buffer for Western blotting analysis.

The GFP-hAQP7 plasmid and the pEGFP-C1 control plasmid were transfected into 293FT cells as described previously^12^. After 48 hours of transfection, cells were treated with culture medium containing 8% EG, 9.5% DMSO or 0.5 M sucrose for 30 min. The cells were washed once with sterile PBS and fixed with 4% PFA for 20 min at room temperature, followed by three washes with PBS.

### Immunofluorescence and image processing

The fixed oocytes were blocked in 1×PBS containing 5% BSA and 1% saponin (Sigma-Aldrich) for 30 min, followed by incubation with primary antibody at a 1:200 dilution at 4 °C overnight. After washing three times, the oocytes were incubated with Alexa Fluor 488/594 goat anti-rabbit IgG (Invitrogen, Carlsbad, USA) at a 1:400 dilution for 30 min. Oocytes were imaged with an Olympus FV1000 laser-scanning confocal microscope (Olympus, Tokyo, Japan) under a Olympus UPlanSApo 20×/0.75 objective lens. The fluorescence intensity of each image was analysed using Image-J software (U.S. National Institutes of Health). The image signal was obtained by measuring the mean pixel intensity of the cell subtracting by the mean background and then multiplying this value by the area of the cell. Finally, the relative intensity corresponded to the treatment group signal divided by the control signal.

The primary antibodies used for immunofluorescence included goat polyclonal anti-AQP3 antibody (Santa Cruz Biotechnology, Santa Cruz, USA), rabbit polyclonal anti-AQP7 antibody (Santa Cruz Biotechnology), rabbit polyclonal anti-AQP9 antibody (Santa Cruz Biotechnology), rabbit polyclonal anti-CPEB antibody (Abcam, San Francisco, USA), rabbit polyclonal anti-phosphorylated of CPEB (T171) antibody (Epitomics, Burlingame, USA), rabbit polyclonal anti-Aurora A antibody (Epitomics), and rabbit polyclonal anti-phosphorylated Aurora A (T288) antibody (Abcam). Filamentous actin (F-actin) was detected using fluorescein isothiocyanate-conjugated phalloidin (Sigma-Aldrich) diluted in phosphate buffer (50 μg/mL).

Images of fixed 293FT cells were taken with an Olympus FV1000 laser-scanning confocal microscope under a 60×/1.4 oil objective lens. The presented images are representative of 3 experiments. The fluorescence intensities from 20 different cells of one representative experiment were quantified using Image-J software.

### Co-immunoprecipitation

For co-immunoprecipitation, 293FT cells transfected with the GFP-hAQP7 plasmid were lysed in a solution containing (in mM): 25 HEPES, 100 NaCl, 1 EDTA, 0.5 MgCl_2_, 10 NaF, 1 phenylmethylsulphonyl fluoride (PMSF), 1 Na orthovanadate, 1 aprotinin, and 1 leupeptin, plus 10% glycerol and 1% NP-40 (pH 7.5), as previously described^35^. Lysates were put on ice for 30 min and then centrifuged for 20 min at 12,000 g. The supernatant was incubated with mouse anti-GFP (Epitomics) or mouse monoclonal anti-F-actin (Abcam) antibody, 1:200 diluted, at 4 °C overnight and then precipitated with protein A/G agarose beads (Merck & Co, Kenilworth, USA). The precipitates were washed three times in lysis buffer and subjected to Western blotting analysis. Antibodies used in Western blot analysis included rabbit polyclonal anti-AQP7 antibody (Santa Cruz Biotechnology) and mouse monoclonal anti-F-actin antibody (Abcam).

### Microinjection of siRNA in mouse oocytes

For knockdown of AQP7 expression, oocytes at the germinal vesicle stage (immature oocytes) were obtained by puncturing follicles on the ovaries of female mice without the injection of hCG, 46–50 h after the injection of PMSG. Mouse oocytes exhibiting a normal appearance were stored at 37 °C in HEPES-buffered HTF containing 10% serum substitute supplement (pH 7.4) covered with mineral oil (Sigma-Aldrich). These oocytes were injected with scrambled RNA (Ambion, Austin, USA), mouse *AQP3* siRNA (S62527, sense: 5′-GGAUUGUUUUUGGGCUGUATT-3′; antisense: 5′-UACAGCCCAAAACAAUCCCA-3′, Ambion) or mouse *AQP7* siRNA (sense: 5′-GCAGCUACCACCUACUUAATT-3′; antisense: 5′-UUAAGUAGGUGGUAGCUGCAG-3′, Ambion) and then cultured until they matured to the metaphase II stage. In microinjection experiments, each oocyte was held with a holding pipette connected to a micromanipulator on an inverted microscope and injected with 10 pl scrambled RNA solution (1 pg/pl, as a control) or with *AQP3* or *AQP7* siRNA solution (1 pg/pl) with an injection needle connected to another micromanipulator. Injected oocytes with a normal appearance were cultured in HTF containing 10% serum substitute supplement, 0.1 IU/ml FSH, 0.5 IU/ml HCG and 0.05 mg/ml penicillin under 5% CO_2_ at 37 °C. AQP7 mRNA and protein expression levels were examined 16–18 hours after the injection, using qPCR and immunofluorescence, respectively.

### qPCR

Total RNA was extracted from 30–50 mouse oocytes using the RNeasy Plus Micro Kit according to the manufacturer’s instructions (Qiagen, Hilden, Germany). The cDNA was prepared by reverse transcription, using the RT reagent Kit (Takara, Dalian, China). RT-nested PCR was repeated at least three times.

qPCR was carried out with SYBR-Green premix Ex Taq (Takara) in an Applied Biosystems 7900 Fast PCR System (ABI, Carlsbad, USA), using GAPDH as an internal control. The primers for AQP3 were sense: 5′-AACCCTGCCCGTGACTTTGGA-3′ and antisense: 5′-CGAAGACACCAGCGATGGAACC-3′. The primers for AQP7 were sense: 5′-CCTTGGTTCCGTGGCTCACAT-3′ and antisense: 5′-CAGAGATGCCGCCTGCTACAT-3′. The primers for GAPDH were sense: 5′-CCCCAGCAAGGACACTGAGCAAGAG-3′ and antisense: 5′-GCCCCTCCTGTTATTATGGGGGTC-3′. qPCR was performed in a 10 μl reaction system containing 5 μl SYBR premix Ex Taq, 0.2 μl sense and 0.2 μl antisense primers, 0.2 μl Dye I, 3.4 μl ddH_2_O, and 1.0 μl cDNA. The thermal cycling conditions were: 95 °C for 10 s, 95 °C for 5 s, and 60 °C for 34 s, and for 40 cycles. Data were analysed by the comparative threshold cycle (CT) method.

### Oocyte vitrification and survival rate analysis

Oocytes injected with scrambled, AQP7 siRNA or AQP3 siRNA were vitrified using a two-step media protocol and thawed as described previously^12^,^36^. Briefly, oocytes were placed in cryoprotectant solution I (8% (v/v) EG in HTF medium) for 5 min, followed by placement in cryoprotectant solution II (15% (v/v) EG, 10 mg/ml Ficoll 70 (Pharmacia Biotech, Uppsala, Sweden) and then in 0.5 M sucrose in HTF medium for less than 30 s at room temperature. Oocytes were sealed in a cryovial and stored in liquid nitrogen. The oocytes were washed twice in HTF medium containing 0.5 M sucrose before being cultured again. The survival rate of the thawed oocytes was assessed by examining the appearance of their cytoplasm and plasma membranes under a stereomicroscope (Nikon, Tokyo, Japan) after culturing at 37 °C and 5% CO_2_ for 2 hours. Oocytes showing a clear outline of the plasma membrane and normal size and colour were considered as surviving cells.

### Signalling pathway inhibitor treatment and analysis

The oocytes were randomly divided into five groups and pretreated with the PKC inhibitor staurosporine (15 nM), the PI3K inhibitor LY294002 (25 μM), the MEK inhibitor U0126 (10 μM), the JNK inhibitor SP600125 (25 μM), or vehicle (control) for 10 min. After pretreatment, oocytes were washed four times with HTF. Oocytes were treated with 8% EG in HTF for 20 min, and then fixed immediately. The protein levels of CPEB, pCPEB, Aurora A, pAurora A and AQP7 were analysed with immunofluorescence as described above. On the other hand, 293FT cells transfected with GFP-hAQP7 or GFP alone were treated with the same protocol. Cells were collected and fixed for imaging or lysed in RIPA buffer for Western blotting. Expression levels of GFP-hAQP7 and GFP were analysed by the fluorescence image processing as described above and by Western blotting.

### Western blotting

Treated oocytes and transfected 293FT cells were lysed in 1× RIPA buffer containing protease inhibitors (1 μg/ml PMSF), respectively. Samples were separated using a 10% SDS gel. The separated samples were transferred to a nitrocellulose transfer membrane. After incubating for 1 h with blocking buffer, the membrane was exposed to primary antibody (1:1000) at 4 °C overnight, followed by incubation with secondary antibodies (DyLight 680 or 800, KPL, Gaithersburg, USA) for 2 hours. The membranes were scanned by Odyssey (Li-cor bioscience, Lincoln, USA). The bands were analysed with Quantity One software (Bio-Rad Laboratories, Hercules, USA). The primary antibodies used in this study included rabbit polyclonal anti-phosphorylation of CPEB (T171) antibody (Epitomics), rabbit polyclonal anti-phosphorylation of Aurora A (T288) antibody (Abcam), rabbit polyclonal anti-AQP7 antibody (Santa Cruz Biotechnology) and mouse monoclonal anti-β-actin antibody (Santa Cruz Biotechnology).

### Statistical analysis

All data were normally distributed and are presented as the mean ± SEM, An independent-samples *t* test was used to evaluate the statistical significance between two groups. One-way analysis of variance (ANOVA) and Turkey’s post-hoc tests were used to evaluate the statistical significance of differences between more than two groups. A chi-square test was used to compare the survival rates of oocytes between the two groups. The WINDOWS version of SPSS 16.0 was used for the statistical analysis. P-values less than 0.05 were considered statistically significant.

## Additional Information

**How to cite this article**: Tan, Y.-J. *et al.* Aquaporin7 plays a crucial role in tolerance to hyperosmotic stress and in the survival of oocytes during cryopreservation. *Sci. Rep.* **5**, 17741; doi: 10.1038/srep17741 (2015).

## Supplementary Material

Supplementary Information
