# Supplementary material for: Aquaporin7 plays a crucial role in tolerance to hyperosmotic stress and in the survival of oocytes during cryopreservation
Source: Sci Rep. 2015 Dec 4;5:17741. doi: 10.1038/srep17741 (PMC4669445; doi:10.1038/srep17741)
Supplement: Supplementary Information [file srep17741-s1.pdf]

Aquaporin7 plays a crucial role in tolerance to hyperosmotic stress and in the survival of oocytes during cryopreservation

Ya-Jing Tan[1, 2], Xue-Ying Zhang [3], Guo-Lian Ding[1, 2], Rong Li[1, 2], Li Wang[1, 2], Li Jin[1, 2], Xian-Hua Lin[2, 3], Ling Gao[1], Jian-Zhong Sheng[3, 4], He-Feng Huang[1, 2, 3*]

[1]Center of Reproductive Medicine, the International Peace Maternity and Child Health Hospital, School of Medicine, Shanghai Jiao Tong University, Shanghai, China.

[2]Institute of Embryo-Fetal Original Adult Disease Affiliated to Shanghai Jiao Tong University School of Medicine, Shanghai Jiao Tong University, Shanghai, China.

[3]The Key Laboratory of Reproductive Genetics, Ministry of Education (Zhejiang University), Hangzhou, China.

[4]Department of Pathology & Pathophysiology, School of Medicine, Zhejiang University, Hangzhou, China.

[*]Corresponding authors. He-Feng Huang, M.D. Center of Reproductive Medicine, The International Peace Maternity and Child Health Hospital, School of Medicine, Shanghai Jiao Tong University, Shanghai, China.

Tel: +86-021-64073897;

Fax: +86-021-64474645;

E-mail: huanghefg@hotmail.com

Supplementary material

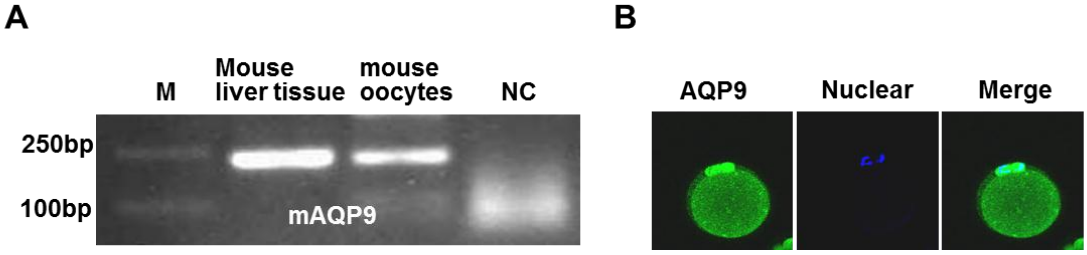

Figure S1. (A) Expression of AQP9 mRNA in mouse oocytes (M: marker; NC: negative control). (B) Immunofluorescence analysis confirmed AQP9 expression in mouse oocytes (green: AQP9; blue: nuclear DNA; scale bar, 20 μm).

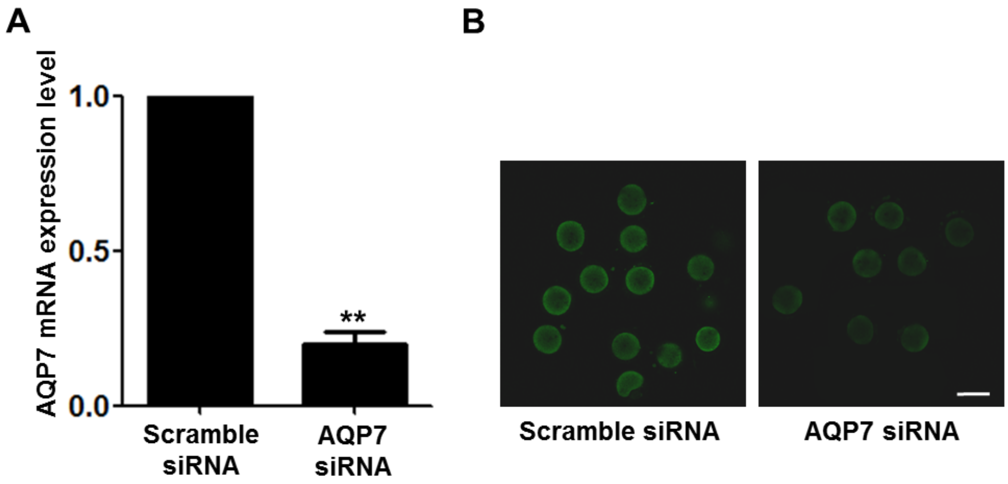

Figure S2. qPCR analysis (A) and immunofluorescence staining (B) show low expression of AQP7 in mouse oocytes injected with AQP7 siRNA. Green: AQP7; scale bar, 100 μm.

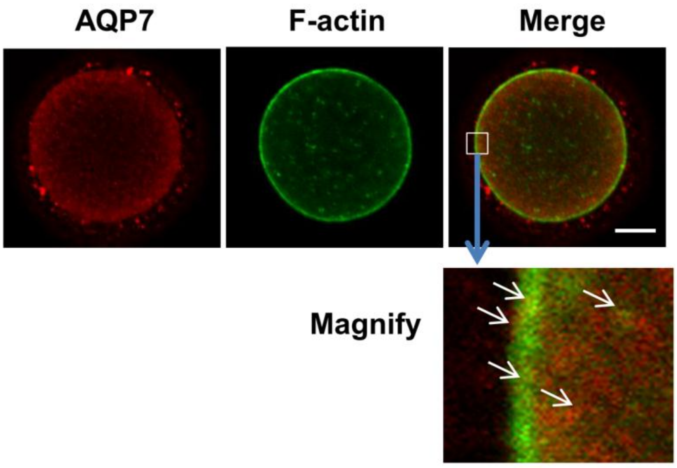

Figure S3. Immunofluorescence staining show AQP7 and F-actin colocalization in mouse oocytes (red: AQP7; green: F-actin). Scale bar, 20 μm. The white arrows show the yellow points which mean colocalization of F-actin with AQP7.

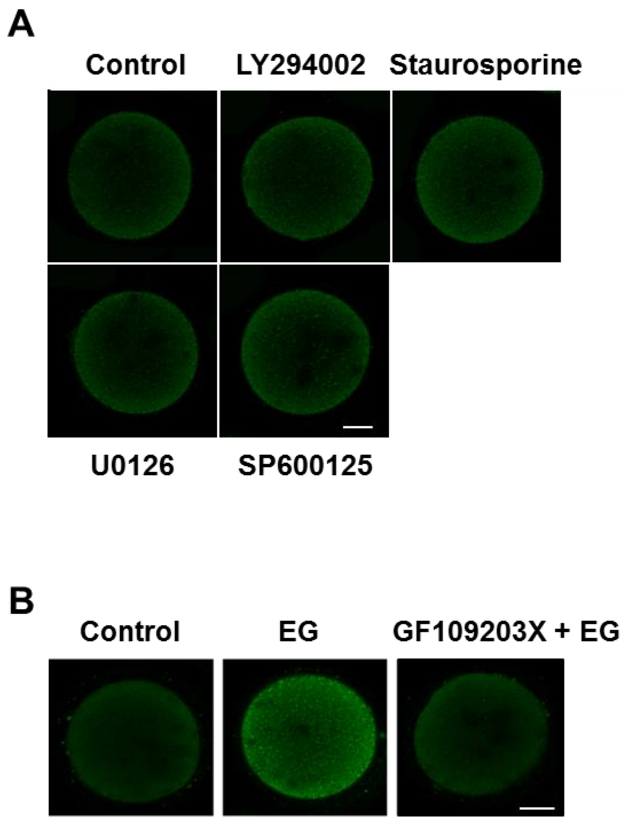

Figure S4. A: Oocytes were pretreated with LY294002, staurosporine, U0126 and SP600125, respectively, and the immunofluorescence intensities of AQP7 were analysed in mouse oocytes without treatment with EG solution. B: Oocytes were pretreated with PKC inhibitor (GF109203X, 20 nM)，and the immunofluorescence intensities of AQP7 were analysed in mouse oocytes in the presence of 8% EG. Control is vehicle. Green: AQP7; scale bar, 20 μm.

Raw data related to western blots of different figs.

Fig. 2D

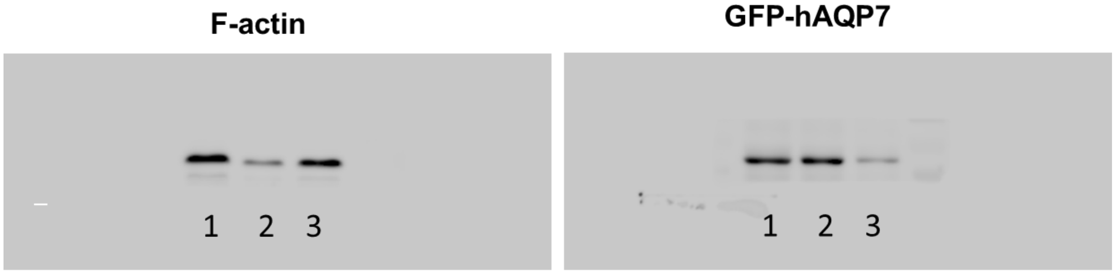

1: Input; 2: IP-GFP; 3: IP-F-actin

Fig. 3E

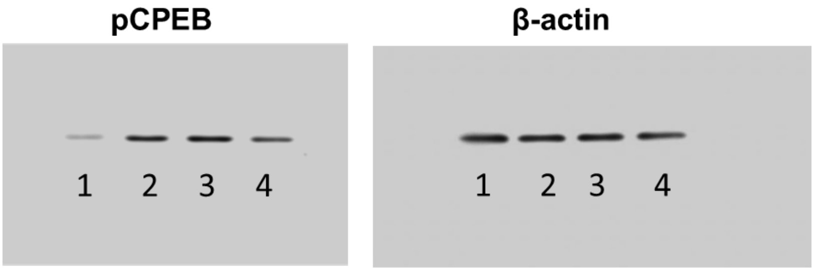

1: Control; 2: EG; 3: DMSO; 4: Sucrose

Fig. 4E

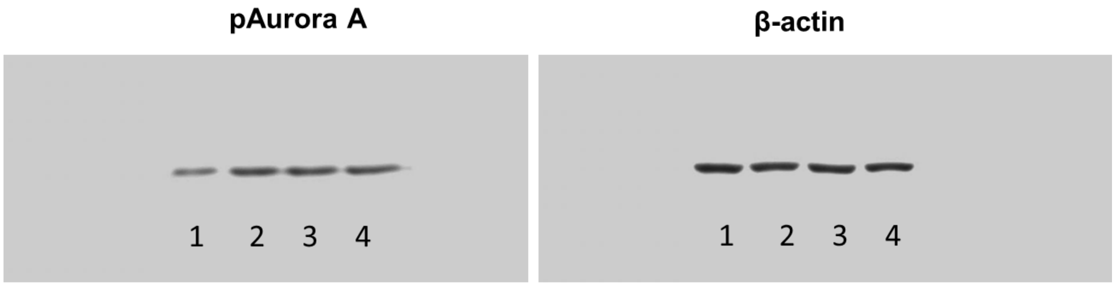

1: Control; 2: EG; 3: DMSO; 4: Sucrose

Fig. 6E

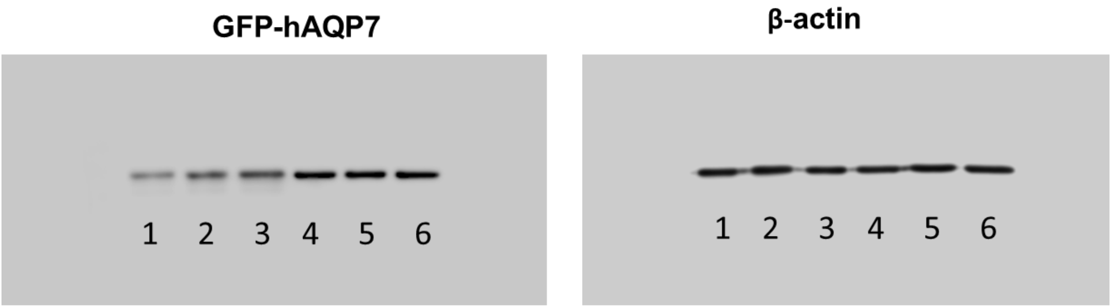

1: Control; 2: LY294002; 3: Staurosporine;
4: U0126; 5: SP600125; 6: EG
